# Differential effects of *Akkermansia*-enriched fecal microbiota transplant on energy balance in female mice on high-fat diet

**DOI:** 10.3389/fendo.2022.1010806

**Published:** 2022-10-27

**Authors:** Kalpana D. Acharya, Randall H. Friedline, Doyle V. Ward, Madeline E. Graham, Lauren Tauer, Doris Zheng, Xiaodi Hu, Willem M. de Vos, Beth A. McCormick, Jason K. Kim, Marc J. Tetel

**Affiliations:** ^1^ Neuroscience Department, Wellesley College, Wellesley, MA, United States; ^2^ University of Massachusetts Chan Medical School, Worcester, MA, United States; ^3^ Center for Microbiome Research, Department of Microbiology and Physiological Systems, University of Massachusetts Chan Medical School, Worcester, MA, United States; ^4^ University of Massachusetts Chan Medical School, Worcester, MA, United States; ^5^ Laboratory of Microbiology, Wageningen University, Wageningen, Netherlands; ^6^ University of Helsinki, Helsinki, Finland; ^7^ Division of Endocrinology, Metabolism, and Diabetes, Department of Medicine, University of Massachusetts Chan Medical School, Worcester, MA, United States

**Keywords:** gut microbiome, metabolism, estrogens, obesity, estradiol, diabetes

## Abstract

Estrogens protect against weight gain and metabolic disruption in women and female rodents. Aberrations in the gut microbiota composition are linked to obesity and metabolic disorders. Furthermore, estrogen-mediated protection against diet-induced metabolic disruption is associated with modifications in gut microbiota. In this study, we tested if estradiol (E2)-mediated protection against obesity and metabolic disorders in female mice is dependent on gut microbiota. Specifically, we tested if fecal microbiota transplantation (FMT) from E2-treated lean female mice, supplemented with or without *Akkermansia muciniphila*, prevented high fat diet (HFD)-induced body weight gain, fat mass gain, and hyperglycemia in female recipients. FMT from, and cohousing with, E2-treated lean donors was not sufficient to transfer the metabolic benefits to the E2-deficient female recipients. Moreover, FMT from lean donors supplemented with *A. muciniphila* exacerbated HFD-induced hyperglycemia in E2-deficient recipients, suggesting its detrimental effect on the metabolic health of E2-deficient female rodents fed a HFD. Given that *A. muciniphila* attenuates HFD-induced metabolic insults in males, the present findings suggest a sex difference in the impact of this microbe on metabolic health.

## Introduction

Loss of estrogens during menopause causes weight gain, resulting in an increased risk of metabolic, cardiac, inflammatory, osteopathic, and neurological disorders (1–4). Protection against diet-induced obesity, hyperglycemia, hyperlipidemia, and insulin resistance are mediated by estrogens in women and in female rodents (5–9). E2-dependent protection against HFD-induced obesity is associated with increased physical activity and basal energy expenditure and improvements in systemic insulin sensitivity and glucose metabolism (7, 9).

Gut microbiota profoundly impact host metabolism. Metabolic syndrome, characterized by adiposity, hyperlipidemia and hyperglycemia, is associated with changes in gut microbiota (10–12). Manipulation of gut microbiota, by depletion *via* antibiotics (12–16), administration of specific bacteria (17–20), or transplantation of fecal/caecal microbiota (14, 21, 22) can improve metabolism, in male rodents and men. In particular, the abundance of bacteria belonging to the Verrucomicrobia and its dominant intestinal genus A*kkermansia*, are negatively associated with obesity in men and women (23–26). *Akkermansia muciniphila* is a mucin utilizer and producer of short chain fatty acids, which have anti-inflammatory properties and are primary nutrients for intestinal endocrine cells (27–29). Moreover, *A. muciniphila* produces signaling proteins, such as the 33-kD Amuc_1100, that interact with the Toll-like 2 receptor and improve barrier function (19, 30).

In male mice fed a HFD, *A. muciniphila* supplementation attenuated obesity and inflammation and improved insulin signaling (17, 19, 31, 32). Only a few studies have investigated the effects of *Akkermansia* in women. In postmenopausal women, *Akkermansia* was negatively correlated with insulin resistance and dyslipidemia (33). Furthermore, heat-killed *A. muciniphila* administration attenuated body weight, fat mass, and hip circumference in obese women and men (18).

There is increasing evidence that estrogens can influence gut microbiota (34–36). Postmenopausal women have higher *Prevotella* and lower *Lachnospira* and *Roseburia* relative abundances, and a lower Firmicutes/Bacteroidetes ratio, when compared to premenopausal women (37). The differences in gut microbiota were attenuated between postmenopausal women and men, and between gonadectomized male and female rats, although baseline sex differences in gut microbiota persist even after the depletion of gonadal estrogens (37, 38). It is possible that these sex differences start during puberty as girls were found to develop towards an adult microbiota earlier than boys (39). Intake of phytoestrogens in women was found to increase beneficial microbes including *Lactobacillus, Enterococcus* and *Bifidobacterium* (40, 41). Ovariectomy or E2 treatment, in wild-type as well as *ob/ob* (leptin-deficient) mice, altered gut microbiota (7, 42–45). Interestingly, while HFD or a high-fat high-sugar diet decreased relative *Akkermanisa* levels in male mice (17, 31, 46), these were increased in HFD-treated female mice, with a further increase in E2-treated groups (7). This sex difference in *Akkermansia* modulation in response to a change in diet indicates a critical need to examine the functions of gut microbiota, including *Akkermansia* supplementation, on female metabolic health. Therefore, in this study, using female mice, we tested if fecal microbiota transplantation (FMT) from E2-treated lean mice, with or without *A. muciniphila* supplementation, protects E2-deficient mice against HFD-induced metabolic insults.

## Materials and methods

Animal experiments were performed at the University of Massachusetts Chan Medical School and Wellesley College. All procedures were approved by the Institutional Animal Care and Use Committees of University of Massachusetts Chan School and Wellesley College and performed in accordance with National Institutes of Health Animal Care and Use Guidelines.

### Animals

Ten-week-old female C57BL/6J mice were housed 3-4/cage on a 12h light-dark cycle, with *ad libitum* food and water. Mice were ovariectomized and silastic capsules filled with 17β-estradiol (E2, 50 μg in 25 μl of 5% ethanol/sesame oil), or vehicle (Veh, 25 μl of 5% ethanol/sesame oil) were subcutaneously implanted as described previously (6, 7, 44, 47).

### Diet

Both donor and recipient female mice were fed phytoestrogen-free standard chow (13% kCal from fat, LabDiet, #5V75R) until they were switched to HFD containing 60% kCal fat (#D12492, Open Sources Diet, USA) for the remainder of the study.

### Experiment 1: Fecal microbiota transplantation and cohousing study: Impact on metabolism

#### Antibiotic administration

To allow an efficient colonization of the donor microbiota by initially depleting native microbiota, all recipients were administered an antibiotic cocktail of ampicillin (1 g/L; #A0166, Sigma-Aldrich, USA), vancomycin (500 mg/L; #PHR1732, Sigma-Aldrich, USA) neomycin (1 g/L; #N6386, Sigma-Aldrich, USA), and metronidazole (1 g/L; #M3761, Sigma-Aldrich, USA) (AVNM), as described previously (48), for a total of 9 days in drinking water. This antibiotic cocktail regimen has been shown to be effective in reducing up to 90% of the native bacterial community and depletes most groups of microbes (*e.g.*, gram positive, gram negative and anaerobes) in male and female mice (49–51).

#### Fecal microbiota transplantation

**Figure 1:**
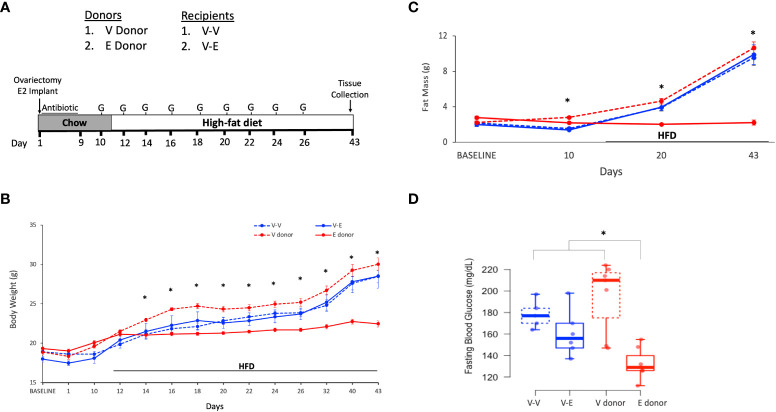
Estradiol treatment but not fecal microbiota transplant (FMT), attenuated obesity and hyperglycemia in female mice on HFD. **(A)**: FMT and cohousing study design and timeline. Mice were fed phytoestrogen-free chow till D11, then switched to HFD. Mice treated with E2 (E donor, n = 7) or Veh (V donor, n = 7) were used as FMT donors. All recipients received Veh implants. V-V and V-E recipients got FMT from V donors or E donors, respectively. Recipient mice were administered antibiotic cocktail for 9 days and starting on D10, were orally gavaged with 9 doses of FMT, on alternate days. G denotes FMT gavage days. **(B)** Body weight; **(C)** Fat mass. For **(B, C)**, repeated measures ANOVA, separately on recipients and donors, followed by a t-test. *denotes p < 0.05 between E2 donors and Veh donors). **(D)** 5h-fasting blood glucose on D19, after one week on HFD. Two-way ANOVA followed by Tukey *post-hoc* (*p < 0.05). V donor, mice with Veh implants used as FMT donors; E donor, E2-treated mice used as FMT donors; V-V, Veh mice receiving FMT from V donor; V-E, Veh mice receiving FMT from E donor.

Fresh fecal samples from mice of the same treatment group were collected and pooled on the morning of the gavage, as described previously (48). FMT was diluted in PBS buffer (0.01M) reduced with 0.5% L-cysteine HCl (1:10), in an anaerobic chamber. Anaerobic environment was created by purging with gas mix (5% H2/10% CO2/85% N) 2-3 times, until the chamber gas reading was 3% H2 and 0 ppm O2. Up to 500µL of reduced PBS buffer was added to 10-15 fecal pellets and gently lysed until no visible pieces were present. FMT mixture was filtered using 70 µm mesh filter and diluted in PBS buffer to bring to a final concentration of 100 mg/ml. Following antibiotic treatment, recipients orally gavaged with 150 µL of 100mg/mL FMT (52, 53). FMT was started on D10 and continued for a total of 9 doses (**Figure 1A**). The days of FMT for the recipients were matched to the days of the fecal sample collection from the donors (*e.g*. on D12, the recipients received FMTs from fecal samples that were collected on D12 from the donors). V-V and V-E recipients received FMT gavage from Veh and E2 donors, respectively.

#### Metabolic measurements

Body weight and body composition (lean/fat mass, using ^1^H-MRS) were measured throughout the study (**Figure 1A**). Blood glucose levels during a five-hour fasting period were measured during chow feeding (D9), a week after the start of HFD (D19) and at the end of the study (D43), to assess the effect of transplanted gut microbiota on glucose homeostasis.

#### Statistical analysis

The effects of E2 and FMT on HFD-induced longitudinal metabolic changes, starting on D10 (1^st^ FMT gavage day), including body weight, fat mass, and lean mass were separately analyzed by a two-way repeated measures ANOVA, followed by a Student’s *t*-test for the days when an effect was present (Jamovi, v 1.8.4.0). A two-way ANOVA followed by a Tukey’s HSD *post-hoc* was used to measure the effects of E2 and FMT on blood glucose levels across groups.

### Experiment 2: *A. muciniphila*-enriched FMT: Impact on metabolism and gut microbiota

#### Diet and antibiotic administration

**Figure 2:**
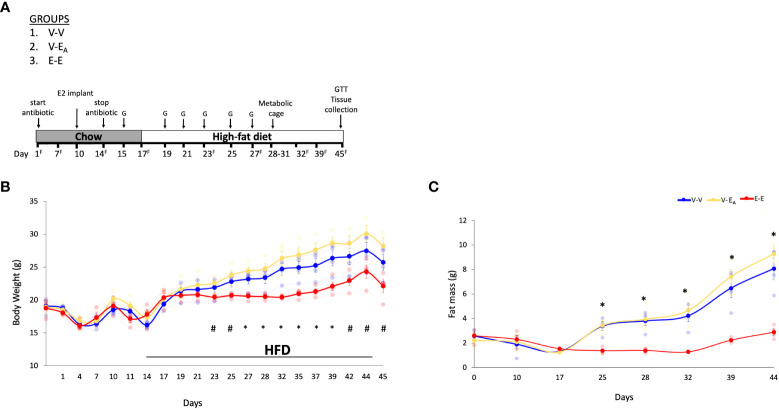
Estradiol treatment, but not fecal microbiota transplant (FMT) from lean E2-treated mice supplemented with *A*. *muciniphila*, protected ovariectomized mice from HFD-induced obesity. **(A)** *A*. *muciniphila*-enriched FMT study design and timeline. Recipient mice were administered antibiotic cocktail for the first 14 days. The three recipient groups, 1) Veh mice receiving FMT from Veh mice (V-V, n = 4); 2) Veh mice receiving FMT from E2 mice following enrichment with *Akkermansia* cells (V-E_A_, n = 4), and 3) E2-implanted mice receiving FMT from E2 mice (E-E, n = 4) were orally gavaged with a total of 6 gavages on alternate days, starting on D15 and excluding D17. G denotes FMT gavage days; ^F^ denotes fresh fecal sample collection days. **(B)** Body weight and **(C)** Fat mass. *denotes days when E-E groups differ from V-V and V-E_A_; # denotes days when E-E differ from V-E_A_ only (p < 0.05, RM ANOVA, Tukey *post-hoc*). V-V: mice with Veh implants receiving FMT from Veh mice; E-E: E2-treated mice receiving FMT from E2 mice; V-E_A_: mice with Veh implants receiving FMT from E2-treated mice with *A*. *muciniphila* supplementation.

Recipient mice were treated as described above except that the AVNM cocktail was administered for 14 days (49, 51, 54), and HFD was introduced on D14 (**Figure 2A**).

#### *A. muciniphila*-supplemented FMT preparation and administration

A *muciniphila* Muc^T^ (ATTC BAA-835) cells were grown in a synthetic medium containing 16 g/l soy-peptone, 4 g/l threonine, and a mix of glucose and N-acetylglucosamine (25 mM each) under strictly anaerobic conditions (19). Cells were then washed in reduced PBS with 25% (vol/vol) glycerol and immediately frozen at −80°C. Within two hours prior to the gavage, fecal pellets were lysed in reduced 0.01M PBS buffer (containing 0.05% L-cysteine HCl) in anaerobic chamber, as described above in Experiment 1 (19). For transfer to the experimental laboratory, the cells were shipped in dry ice, and upon receipt, were quickly aliquoted in smaller volumes for daily gavages on ice under strict anaerobic conditions and stored at -80 C.

Within two hours prior to each gavage, FMT were prepared as described above. *A. muciniphila* preparations were thawed on ice and immediately mixed with the fresh FMT. V-E_A_ recipients (n=4) received 150 μl of oral gavage containing 40mg/mL of FMT supplemented with 2 × 10^8^ *A. muciniphila*. *A. muciniphila*-supplemented FMT were started on D15 and gavaged every other day for a total of 6 doses (**Figure 2A**). Control V-V (n=4) and E-E (n=4) mice were similarly gavaged with FMT from Veh or E2 mice, respectively, without *A. muciniphila* cells supplementation.

#### Metabolic phenotyping

To assess the HFD-induced metabolic changes as an effect of FMT from lean E2-treated mice supplemented with A. muciniphila, food and water intake, respiration, energy expenditure, and locomotor activity were measured in awake mice after 10 days on HFD (D28-D31; **Figure 2A**) using metabolic cages (TSE Systems, Germany), as described previously (7, 55). Resting energy expenditure (EE) and respiratory exchange ratio (RER) were derived from O_2_ consumption and CO_2_ production data. Body weight and body composition (lean/fat mass) were measured throughout the study using ^1^H-MR spectroscope (EchoMRI, Houston, TX, USA).

#### Measurement of glucose homeostasis

Fasting blood glucose was measured weekly starting with HFD feeding and FMT administration. On day 45 following overnight fasting, a glucose tolerance test (GTT) was performed to measure insulin sensitivity. In brief, 20% glucose at 1g/kg BW was injected i.p. and glucose measurements were taken at 0, 15, 30, 60, 90, and 120 mins following injection. Mice were euthanized immediately following GTT.

#### Fecal DNA extraction

Fecal samples were collected from donor and recipient mice throughout the study to confirm the microbial transfer in recipients and to examine the association between gut microbiota and host metabolism. Fresh fecal samples were collected, immediately frozen in dry ice, and stored at -80**°**C. Total DNA was extracted from fecal pellets using the DNeasy PowerSoil Kit (Cat #12888, Qiagen, USA) following the manufacturer’s protocol.

#### Microbial 16S rRNA gene sequencing

##### 16S rDNA community profiling

Microbiome community profiling of fecal DNA was performed by 300nt paired-end 16S rRNA gene sequencing of the V3-4 region on the Illumina MiSeq platform as described (56). The UPARSE/SINTAX pipeline (usearch v10.0.240_i86linux6, rdp_16s_v18.fa) (57) was used to define OTUs and assign taxonomic classifications.

##### Statistical analysis

###### Metabolic data

To examine the combined effects of *Akkermansia* enrichment and FMT from lean E2-treated mice on HFD-induced metabolic changes, longitudinal data starting on D17 (after the first FMT and the start of HFD), including body weight, fat mass, lean mass, blood glucose, and glucose tolerance test (GTT) were analyzed, as described above in Experiment 1. Data from metabolic cage experiments were analyzed using one way ANOVA, or ANCOVA using body weight immediately prior to the metabolic cages as a covariate (VO2 and VCO2), followed by a Tukey posthoc test.

###### Microbial data analysis

Prior to analysis, OTUs which failed to classify to at least the taxonomic Family level were removed to reduce spurious OTUs. Abundances were summed according to assigned taxonomic classifications for analysis at higher taxonomic levels. Analysis was conducted using either QIIME2 (ver. 2021.4) (58) or MaAsLin2 (ver. 1.8.0) (59) as appropriate. For multiple comparisons, FDR corrections were done and q<0.1 was considered significant.

## Results

### Experiment 1: Effect of FMT and cohousing on energy metabolism in female mice

#### E2 treatment prevented body weight gain, fat mass gain, and hyperglycemia in adult female mice

In order to assess the effects of E2 on body composition, ovariectomized mice receiving implants of estradiol (E2, n=7) or vehicle (Veh, n=7), were analyzed for changes in body weight and fat mass. In contrast to the recipient mice (below), these animals served as E2 donors and Veh donors, and did not receive antibiotic treatment.

E2 prevented weight gain over 6 weeks (**Figure 1B**). Veh groups weighed more than E2-treated mice after 3 days on HFD (D14) through the end of the study (**Figure 1B**). Moreover, E2 attenuated fat mass gain compared to Veh mice (E-donor and V-donor, respectively; **Figure 1C**). As reported previously (7) lean mass was not affected by E2 treatment (data not shown). E2 also prevented hyperglycemia in mice fed HFD for a week (**Figure 1D**), with this effect maintained after 4 weeks on HFD (after 6 weeks of E2 implant; T-test, p<0.001; 95%CI [-77.3, -40.4].

#### FMT and cohousing does not protect Veh animals from HFD-induced obesity

Ten-week old ovariectomized C57BL/6J recipient mice were divided into 2 groups: 1) Veh implanted recipients that received FMT from V-donor mice (V-V, n=5) and 2) Veh implanted recipients that received FMT from E2-treated donor mice (V-E, n=6). In addition to the FMT, V-V and V-E recipients were cohoused with Veh and E2 mice, respectively, at a ratio of 1:1 to transfer microbiota *via* coprophagy (60), starting on the last day of antibiotic treatment (**Figure 1A**). FMT did not protect recipient against HFD-induced body weight and fat mass gain (**Figures 1B, C**). While FMT and cohousing with E2-treated lean mice did not affect blood glucose levels, a trend (p=0.07, one-tailed t-test) towards a decrease was detected in V-E recipients compared to V-V on D19 (**Figure 1D**).

### Experiment 2: Effect of *A. muciniphila*-supplemented FMT on metabolism and gut microbiota

Ten week-old female ovariectomized C57BL/6J E2- or Veh-implanted mice (n=7/group)were group-housed with 3 mice/cage. As in the Experiment 1, the FMT gavage days in recipients were matched with the fecal sample collection days in donors. Recipients were divided into 3 groups with: 1) Veh implants receiving FMT from Veh mice (V-V; n=4), 2) Veh implants receiving FMT from E2-treated mice supplemented with *A. muciniphila* cells immediately prior to gavage (V-E_A_; n=4) and 3) E2 implants receiving FMT from E2-treated mice (E-E; n=4) (**Figure 2A**). FMT from E2-treated lean mice supplemented with *A. muciniphila* did not prevent body weight and fat mass gain in ovariectomized mice.

E-E mice gained less body weight (**Figure 2B**) and fat mass (**Figure 2C**) compared to both Veh groups, V-V and V-E_A_. Longitudinal analysis showed that E-E mice weighed less than V-E_A_ mice from D23 and through the rest of the study (**Figure 2B**). Similarly, E-E mice weighed much less than V-V mice, on D27, D29 and D32-D35 (p<0.05; **Figure 2B**). Compared to the E-E group, the weight gain in the V-V and V-E_A_ mice was mostly due to fat weight starting on D25 (**Figure 2C**).

Unlike E2 treatment, FMT from E2-treated lean mice supplemented with *A. muciniphila* did not prevent weight gain or fat mass in recipient females. While the body weight of V-E_A_ mice tended to be slightly higher than V-V controls a week after the last FMT (**Figure 2**), this effect was not significant (p=0.16). As in Experiment 1 described above, lean mass was not affected by E2 treatment or FMT.

#### E2 treatment improved glucose homeostasis in female mice, but *A. muciniphila*-supplemented FMT from E2-treated lean mice impaired glucose homeostasis

**Figure 3:**
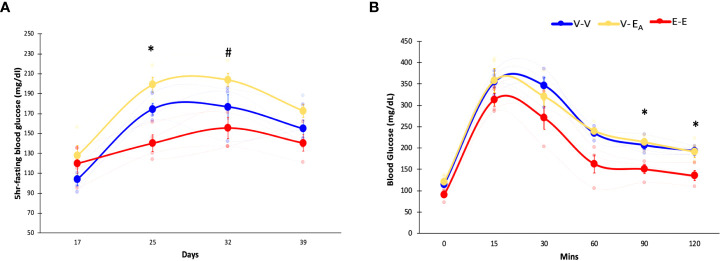
Estradiol treatment protected, while *A*. *muciniphila*-supplemented fecal microbiota transplant (FMT) from E2-treated lean mice exacerbated, HFD-induced hyperglycemia in female mice. **(A)** Blood glucose levels during HFD feeding, **(B)** Glucose tolerance test (GTT) measured on D45. *denotes differences across E-E, V-E_A_, and V-V. # denotes differences between E-E and V-E_A_ (*, # p < 0.05, t-test). E-E, E2-treated mice receiving FMT from E2 mice (n = 4); V-V, mice with Veh implants receiving FMT from Veh mice (n = 4); V-E_A_, mice with Veh implants receiving FMT from E2-treated mice supplemented with *A*. *muciniphila* (n = 4).

Fasting blood glucose levels were measured weekly in recipient mice during HFD feeding. E-E mice had lower blood glucose compared to both V-E_A_ and V-V groups on D25, and V-E_A_ on D32 (**Figure 3A**), indicating protection from HFD-induced hyperglycemia.

To assess the effects of E2 on insulin sensitivity, GTT was measured in recipients following overnight fasting and injection of 20% glucose (*i.p.*, 1g/kg body weight) (61). GTT blood glucose was increased in both V-V and V-E_A_ groups compared to E-E mice at 90 and 120 mins following injection (**Figure 3B**).

Unlike E2 treatment, FMT from E2 mice supplemented with *A. muciniphila* increased fasting glucose levels in V-E_A_ compared to V-V controls on D25 (p<0.05, t-test; **Figure 3A**), suggesting a negative impact of FMT supplemented with *Akkermansia* on glucose homeostasis in female mice. However, *A. muciniphila*-supplemented FMT had no effect on GTT glucose levels (**Figure 3B**).

#### E2 treatment, but not *A. muciniphila*-supplemented FMT from E2-treated lean mice, attenuated energy intake and expenditure in HFD-fed female mice

**Figure 4:**
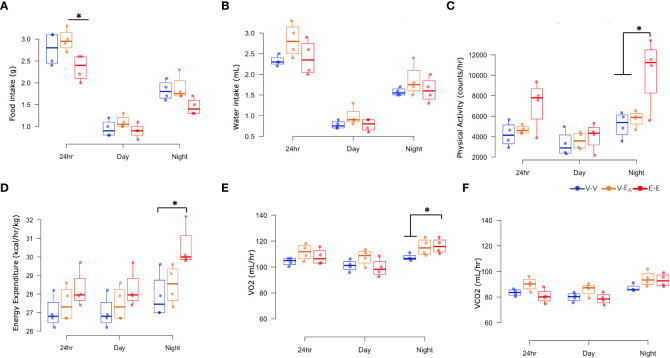
Estradiol, but not fecal microbiota transplant (FMT) from E2-treated mice supplemented with *A*. *muciniphila*, protected against changes in energy intake and expenditure in HFD-fed female mice. **(A)** Food intake, **(B)** Water intake, **(C)** Physical activity, **(D)** Energy expenditure, **(E)** VO2 consumption, and **(F)** VCO2 production were measured in metabolic cages on D18–21 **(A–D)**: *p < 0.05, ANOVA, Tukey *post-hoc*; **(E)**: *p < 0.05, ANCOVA, Tukey *post-hoc*). E-E, E2-treated mice receiving FMT from E2 mice (n = 4); V-V, mice with Veh implants receiving FMT from Veh mice (n = 4); V-E_A_, mice with Veh implants receiving FMT from E2-treated mice supplemented with *A*. *muciniphila* (n = 4).

To test if E2-mediated protection against HFD-induced obesity is associated with energy intake, food and water consumption were measured in female mice, using metabolic cages. E-E groups ate less during 24h and showed a strong trend towards a decrease during night (p=0.062), compared to V-E_A_ groups. E2 increased physical activity during night in E-E mice compared to V-V and V-E_A_ mice. In all treatment groups, food intake and physical activity peaked at 2h after light-off and an hour before the light-on phase (**Supplemental Figure 1**). Additionally, E2 increased basal energy expenditure and VO2 consumption and showed a strong trend towards an increase in VCO2 production (p=0.06) during night, compared to V-V groups (**Figure 4**). Similarly, V-E_A_ groups showed a slight trend towards an increase in energy expenditure compared to E-E mice (p=0.08).

In contrast to E2, FMT from E2-treated animals supplemented with *A. muciniphila* did not improve any metabolic measures. Taken together, these data suggest that E2 regulates energy homeostasis, in part by decreasing energy intake and increasing energy expenditure, whereas FMT has no effect.

#### Antibiotic treatment profoundly decreased gut microbiota in adult female mice

**Figure 5:**
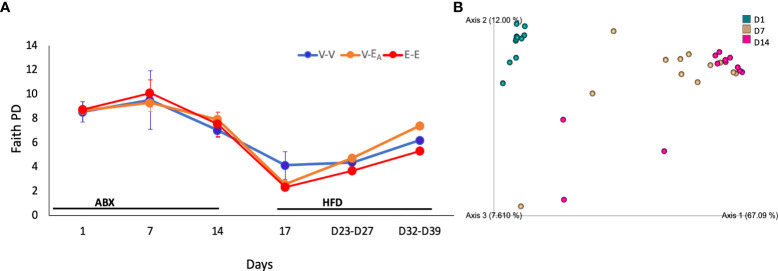
Antibiotics altered gut microbiota *α*-diversity and *β*-diversity in adult female mice. Mice received antibiotics in drinking water for two weeks. Starting on D15, mice received a total of 6 doses of FMT gavage with (V-E_A_) or without *A*. *muciniphila* (E-E and V-V) supplementation. **(A)** Faith phylogenetic diversity (PD) (± *SEM*) over time. Gut microbiota data from D23 and D27 (n = 8) were aggregated to isolate the effect during FMT, and D32 and D39 (n = 8), after the FMT was discontinued. **(B)** Principal component plot showing weighted Unifrac distance between microbiota communities during antibiotic treatment. E-E, E2-treated mice receiving FMT from E2 mice; V-V, mice with Veh implants receiving FMT from Veh mice; V-E_A_, mice with Veh implants receiving FMT from E2-treated mice, supplemented with *A*. *muciniphila*.

Fecal DNA was used to generate 16S rRNA amplicons that were sequenced at 0, 1 and 2 weeks to examine the effect of the two week-long antibiotic treatment. A longitudinal analysis of gut microbiota during antibiotic treatment showed an effect of time (F_(2,20)_=15, p=0.036) on diversity (Faith’s PD (62). Specifically, D14 diversity differed from D1 (ANOVA, p=0.038) and showed a trend towards a decrease compared to D7 (ANOVA, p=0.06; **Figure 5A**). Similarly, β-diversity (weighted UniFrac), showed that the D1 microbiome was significantly different from D7 and D14 (q=0.001; pairwise PERMANOVA) (**Figure 5B**; **Table 1**), where the effect of antibiotic was explained by principal component 1(67%). Taken together, these results confirm that antibiotics deplete microbial community as early as within one week of the treatment.

**Table 1:** Taxa that differed as an effect of antibiotics, across D1, D7 and D14 (n=4/per group).

Features	N	N.not.0	coef_D7	stderr-D7	pval_D7	qval_D7	coef_D14	stderr-D14	pval_D14	qval_D14
g:Bacillus	35	23	5.502631789	0.90805299	9.13E-07	2.59E-05	8.0183231	0.90805299	4.34E-10	7.38E-08
g:Lachnospiracea_incertae_sedis	35	17	-4.692415702	0.79195076	1.35E-06	2.86E-05	-5.5974098	0.79195076	5.14E-08	2.91E-06
g:Staphylococcus	35	20	7.689429355	1.361851202	3.75E-06	5.79E-05	4.87725576	1.3618512	0.001188009	0.007189637
g:Coprococcus	35	9	-1.608431721	0.321681427	1.99E-05	0.000241245	-1.6969855	0.32168143	8.92E-06	0.00011662
g:Brevibacillus	35	19	6.295611124	1.302246091	3.21E-05	0.000364206	5.08950002	5.08950002	1.302246091	0.000452906
g:Paenibacillus	35	21	4.091524263	0.869358639	4.66E-05	0.00046561	5.69927166	0.86935864	2.19E-07	9.32E-06
g:Anaerotruncus	35	16	-4.009935921	0.856886741	5.73E-05	0.000541573	-5.5095071	0.85688674	4.20E-07	1.43E-05
g:Clostridium_IV	35	25	-5.107525024	1.143259238	9.27E-05	0.000750319	-8.2361222	1.14325924	3.51E-08	2.91E-06
g:Rhizobium	35	17	4.28364346	0.985040526	0.000130281	0.001006719	3.20692547	0.98504053	0.002675368	0.012282122
g:Streptococcus	35	17	4.25052927	1.071323406	0.000383635	0.002608719	3.48671909	1.07132341	0.002682695	0.012282122
g:Oscillibacter	35	25	-3.401412103	0.917564998	0.000847488	0.005336038	-5.4710768	0.917565	1.54E-06	2.91E-05
g:Thermobacillus	35	14	5.220228325	1.48112452	0.001303312	0.007385434	3.4168798	1.48112452	0.027681541	0.069203852
g:Clostridium_XlVa	35	32	-3.857938046	1.189210373	0.002757749	0.012282122	-5.0736799	1.18921037	0.000164763	0.001217814
g:Lactococcus	35	13	3.669877108	1.114432971	0.002542727	0.012282122	2.59318819	1.11443297	0.026900937	0.069005118
g:Saccharopolyspora	35	14	1.991895064	0.629979376	0.003570759	0.014453071	1.72937606	0.62997938	0.010115452	0.036587805
g:Oceanobacillus	35	15	4.164434193	1.359390653	0.004585353	0.018128138	4.68120711	1.35939065	0.001711298	0.00909127
g:Corynebacterium	35	15	2.227561334	0.772514468	0.006976508	0.026355698	2.18637628	0.77251447	0.00797127	0.029459043
g:Enterococcus	35	14	2.949128139	1.234963216	0.023011672	0.063849217	2.92205345	1.23496322	0.024198141	0.063849217
g:Pantoea	35	14	3.356320842	1.419081154	0.024251843	0.063849217	4.58740204	1.41908115	0.00284218	0.01228212
g:Eubacterium	35	18	-1.912312654	0.873924111	0.036574358	0.087572407	-5.2756427	0.87392411	1.26E-06	2.86E-05
Taxa that differed from D1 with D7 only										
g:Romboutsia	35	12	3.141377684	0.885668808	0.001226468	0.007189637				
g:Bifidobacterium	35	8	3.988014783	1.139138124	0.001389308	0.007618788				
g:Akkermansia	35	33	4.420231672	1.358572488	0.002806638	0.012282122				
g:Aneurinibacillus	35	7	3.164196162	0.975453827	0.002889911	0.012282122				
g:Devosia	35	7	1.598777515	0.594557025	0.011580032	0.041012614				
g:Virgibacillus	35	6	1.621198268	0.628616619	0.014710939	0.050017192				
g:Anaerosalibacter	35	8	1.311939773	0.515302298	0.015913519	0.051043363				
g:Ochrobactrum	35	9	2.346538607	0.917315627	0.015777269	0.051043363				
g:Sporosarcina	35	4	0.964934599	0.375930075	0.015481996	0.051043363				
g:Propionibacterium	35	9	1.432460254	0.56690916	0.016652362	0.052424102				
g:Peptoniphilus	35	4	1.593208679	0.663970382	0.022411279	0.063849217				
g:Thermoactinomyces	35	7	1.910299743	0.808680992	0.024412936	0.063849217				
g:Tissierella	35	4	1.589303808	0.65536978	0.021514157	0.063849217				
										
Taxa that differed from D1 with D14 only										
g:Clostridium_XlVb	35	25	-5.892136257	1.038177518	3.45E-06	5.79E-05				
g:Acetatifactor	35	24	-3.628957577	0.652050595	4.67E-06	6.61E-05				
g:Anaeroplasma	35	27	-7.181276595	1.503504437	3.80E-05	0.00040401				
g:Parasutterella	35	31	-5.653802552	1.221706283	6.65E-05	0.000594602				
g:Turicibacter	35	28	-6.349730542	1.379395374	7.08E-05	0.000601784				
g:Prevotella	35	14	3.486544325	0.821138046	0.000192928	0.00136657				
g:Ureibacillus	35	8	3.124919996	0.91934495	0.001827382	0.009413787				
g:Bacteroides	35	33	-3.020058125	0.939049582	0.003102949	0.012865885				
g:Erysipelotrichaceae_incertae_sedis	35	12	-2.4032086	0.79130475	0.00490704	0.018959				
g:Faecalibacterium	35	7	2.80497193	1.08362548	0.01438048	0.04989147				
g:Streptomyces	35	11	2.40347418	0.96770209	0.01842974	0.05696465				
g:Asaccharobacter	35	8	-1.2761367	0.52196992	0.020179	0.06125768				
g:Acinetobacter	35	4	0.668838904	0.283000347	0.024349165	0.063849217				
g:Anaerofustis	35	6	-1.040876644	0.438486716	0.023775938	0.063849217				
g:Mycobacterium	35	8	1.720756696	0.725809859	0.023939009	0.063849217				
g:Clostridium_sensu_stricto	35	33	-3.309072706	1.425391057	0.027196135	0.069005118				
g:Methylobacterium	35	10	1.46379455	0.64507776	0.03013663	0.07424967				
g:Butyricicoccus	35	10	-1.6990275	0.76372172	0.03327949	0.08082163				
g:Ruminococcus	35	11	-1.6825484	0.78762838	0.04040904	0.09541024				

Antibiotics were administered from D1 to D14. q<0.1 were considered significant. Coefficients represent the magnitude and the direction of change on the days specified.

**Figure 6:**
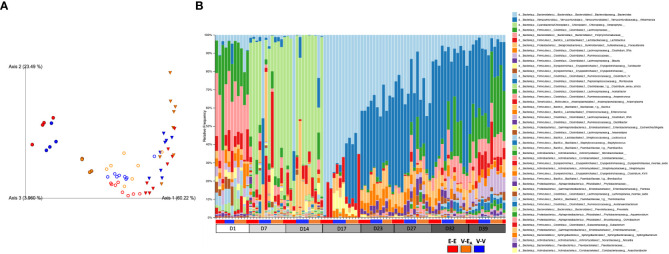
Estradiol, antibiotics, and fecal microbiota transplant (FMT) from E2-treated lean mice supplemented with *A*. *muciniphila* altered gut microbiota *α*-diversity and *β*-diversity in adult female mice. Mice were administered antibiotics for two weeks. From D15, mice received 6 total doses of FMT gavage with (V-E_A_) or without (E-E and V-V) *A*. *muciniphila* supplementation. **(A)** Principal component plot showing weighted Unifrac distance for microbial communities during [●: D17; o: D23 and D27 aggregate (n=8)] after FMT [*▽*: D32 and D39 aggregate (n=8)]. **(B)** Microbiota taxa relative abundances at the genus level, across treatment days. E-E, E2-treated mice receiving FMT from E2 mice; V-V, mice with Veh implants receiving FMT from Veh mice; V-E_A_, mice with Veh implants receiving FMT from E2-treated mice, supplemented with *A* *muciniphila*.

The effect of antibiotics on taxonomic abundances was examined by combining the three treatment groups within each day for D1-D14 data and comparing across days. Multiple *Clostridium* species, *Oscillibacter, Coprococcus, Anaerotruncus* and *Eubacterium* were decreased on both D7 and D14 compared to D1. An additional 12 taxa, including *Bacteroides, Anaeroplasma, Turicibacter*, and *Acetatifactor*, were decreased on D14, compared to D1 as an effect of antibiotic treatment (**Table 1**; **Figure 6**). Aberrations in the fecal communities, including increased levels of multiple *Bacillus*, *Rhizobium, Pantoea*, *Corynebacterium*, and *Lactococcus* spp., were observed due to antibiotic treatment on D7 and D14. Thirteen additional taxa, including *Akkermansia, Bifidobacterium, Romboutsia* and *Devosia*, were increased on D7, compared to D1. Other taxa, such as *Prevotella*, *Fecalibacterium, Mycobacterium*, and *Methalobacterium*, were increased on D14 only compared to D1 (**Table 1**; **Figure 6**).

#### FMT from E2-treated lean mice supplemented with *A. muciniphila* altered gut microbiota in female mice

To examine the effects of FMT during chow or HFD feeding, gut microbiota was compared between treatment groups within each diet. First, the presence of *Akkermansia* was confirmed in the FMT samples that were supplemented with A. *muciniphila* before the gavage (**Supplemental Figure 2**). To determine the effects of FMT during chow, gut microbiota from D17 was analyzed. Gut microbiota was profoundly altered on D17, two days after a single dose of the *A. muciniphila* -supplemented FMT in the V-E_A_ group. As expected, this change in microbiota at D17 was primarily due to *A. muciniphila*, with about 30% of the total gut microbiota in V-E_A_ recipients being comprised of *A. muciniphila* (**Figure 6B**). While the α-diversity (Faith PD) did not differ across groups, microbial composition clustered separately between V-E_A_ and V-V mice (PERMANOVA, q=0.037; **Figure 6A** and **Table 2**). The profound increase in *A. muciniphila* in V-E_A_ mice on D17 was accompanied with decreases in *Clostridium_sensu*, *Parasutterella*, and *Bacteroides*, suggesting an increase in *A. muciniphila* in antibiotic-treated mice can negatively impact abundance of other microbes in the gut (**Table 2**).

**Table 2:** Taxa that differed as an effect of *A. muciniphila*-enriched FMT, between V-V and V-E_A_ recipient female mice on different days during and after completion of the FMT.

feature	Day	reference	value	coef	stderr	N	N.not.0	pval	qval
g:Akkermansia	D17	V-EA	V-V	-5.8991747	1.1083155	12	12	0.00047931	0.00311549
g:Clostridium_sensu_stricto	D17	V-EA	V-V	2.92657739	0.62200336	12	8	0.00111218	0.00570907
g:Parasutterella	D17	V-EA	V-V	8.47253758	2.23419856	12	11	0.00426817	0.01585321
g:Bacteroides	D17	V-EA	V-V	0.32188603	0.1099517	12	12	0.01682468	0.0546802
g:Anaerotruncus	D23 & D27	V-EA	V-V	-2.5302253	0.7573276	23	14	0.00325494	0.0176697
g:Erysipelotrichaceae_incertae_sedis	D23 & D27	V-EA	V-V	-3.542254	1.28407512	23	10	0.01211474	0.041057
g:Lactococcus	D32 & D39	V-EA	V-V	-1.3044225	0.48808687	24	24	0.01425087	0.05700348
g:Oscillibacter	D32 & D39	V-EA	V-V	-2.6256781	1.03575443	24	16	0.01925981	0.06676732
g:Desulfovibrio	D32 & D39	V-EA	V-V	-0.8587856	0.36728748	24	5	0.02935066	0.08479079

q<0.1 were considered significant. Coefficients represent the magnitude and the direction of change of the taxa in the “value” groups. V-V, mice with Veh implants receiving FMT from Veh mice (n=4); V-E_A_, mice with Veh implants receiving FMT from E2-treated mice supplemented with *A. muciniphila* (n=4).

Once mice were started on HFD, relative abundance data from the FMT on days 23 and 27 were aggregated to represent the effect during FMT. V-E_A_ recipients had an increase in phylogenetic diversity compared to E-E (ANOVA, Tukey posthoc, p=0.009), but were similar to V-V. Additionally, D23 and D27 microbial community of V-E_A_ mice clustered separately from E-E groups (PERMANOVA, q=0.004; **Figure 6A**). The effect of the FMT was detected on taxa levels as well, such that V-E_A_ mice had higher relative abundances of *Anaerotruncus* and *Erysipelotrichaceae_incertae_sedis* compared to V-V controls (**Figure 6B**; **Table 2**). Of interest, the *Akkermansia* levels increased as expected in the D17 sample of the V-EA mice, consistent with our earlier finding in females (7) but different from findings reported in males (46).

Gut microbiota from the two weeks (D32 and D39) immediately after the last FMT treatment were aggregated and analyzed to capture the late-emerging effects of FMT. In the two weeks post-FMT, V-E_A_ mice had increased α-diversity compared to V-V (p=0.019) and E-E (p<0.001) (**Figure 5A**). Moreover, V-E_A_ microbial community distances differed from both V-V and E-E groups, (PERMANOVA, q=0.003) (**Figure 6A**), suggesting a long-lasting effect of FMT treatment on gut microbiota. On taxa level, V-E_A_ mice showed increased abundances of *Oscillibacter* and *Desulfovibrio* compared to V-V (**Table 2** and **Figure 6B**), suggesting that the effect of FMT continued even after the treatment was ended.

#### Estradiol altered gut microbiota in antibiotic-treated adult female mice fed a HFD

On D17 (a week after E2 treatment), E-E groups clustered differently from V-V and V-E_A_ (PERMANOVA, q=0.037, **Figure 6A**). These differences in communities were due to decreases in relative abundances of *Turicibacter, Parasutterella, Enterococcus*, and *Clostridium_sensu_stricto*, in E-E mice compared to V-V (**Figure 6B** and **Table 3**).

**Table 3:** Taxa that differed as an effect of estradiol treatment, between E-E and V-V recipient female mice on different days.

feature	Day	reference	value	coef	stderr	N	N.not.0	pval	qval
g:Parasutterella	D17	E-E	V-V	9.27240687	2.23419856	12	11	0.00248358	0.00807165
g:Enterococcus	D17	E-E	V-V	10.3067935	0.6352411	12	11	5.69E-08	7.40E-07
g:Turicibacter	D17	E-E	V-V	11.6987047	0.69476585	12	11	4.12E-08	7.40E-07
g:Clostridium_sensu_stricto	D17	E-E	V-V	4.24242391	0.62200336	12	8	7.72E-05	0.00040167
g:Escherichia.Shigella	D23 & D27	E-E	V-V	-8.9731462	0.29054047	23	10	2.37E-18	9.02E-17
g:Anaerostipes	D23 & D27	E-E	V-V	1.96412107	0.3140552	23	23	4.16E-06	5.27E-05
g:Turicibacter	D23 & D27	E-E	V-V	4.17942598	0.91598563	23	13	0.00018903	0.00102615
g:Lactobacillus	D23 & D27	E-E	V-V	3.46545363	0.86845311	23	23	0.00071953	0.00303804
g:Lactococcus	D23 & D27	E-E	V-V	1.8523318	0.49490185	23	23	0.00128263	0.00487401
g:Blautia	D23 & D27	E-E	V-V	1.09027262	0.32260018	23	23	0.00297788	0.00942994
g:Clostridium_XlVb	D23 & D27	E-E	V-V	5.56667665	1.74996858	23	13	0.0046952	0.01274411
g:Clostridium_XlVa	D23 & D27	E-E	V-V	0.92395754	0.30381128	23	23	0.00644792	0.01531381
g:Anaerotruncus	D23 & D27	E-E	V-V	1.91020351	0.73164798	23	14	0.01673041	0.03027407
g:Akkermansia	D23 & D27	E-E	V-V	-0.3032407	0.13064537	23	23	0.03096111	0.05115314
g:Clostridium_IV	D23 & D27	E-E	V-V	1.79460626	0.85996355	23	23	0.04991265	0.07902837
g:Escherichia.Shigella	32&39	E-E	V-V	-4.655963	0.72013279	24	8	2.09E-06	5.43E-05
g:Clostridium_XlVb	32&39	E-E	V-V	5.09111819	1.37522004	24	21	0.00132169	0.01106619
g:Acetanaerobacterium	32&39	E-E	V-V	3.4763462	0.98454697	24	11	0.00198234	0.01171108
g:Parasutterella	32&39	E-E	V-V	-4.9197698	1.40332016	24	24	0.00210334	0.01171108
g:Anaerotruncus	32&39	E-E	V-V	0.96974983	0.323788	24	22	0.00689998	0.02553765
g:Clostridium_sensu_stricto	32&39	E-E	V-V	2.98617404	1.01640665	24	17	0.00785774	0.02553765
g:Enterococcus	32&39	E-E	V-V	1.6602188	0.56047891	24	23	0.00743725	0.02553765
g:Turicibacter	32&39	E-E	V-V	2.89020674	0.97452611	24	19	0.00737629	0.02553765
g:Clostridium_XVIII	32&39	E-E	V-V	3.34725186	1.18261181	24	12	0.01002185	0.03035412
g:Lactobacillus	32&39	E-E	V-V	3.95894107	1.65384916	24	24	0.02609186	0.05575199
g:Romboutsia	32&39	E-E	V-V	4.10493915	1.83282689	24	20	0.03606114	0.07212227

Coefficients represent the magnitude and the direction of change of the taxa in the “value” groups. E-E, E2-treated mice receiving FMT from E2 mice (n=4); V-V, mice with Veh implants receiving FMT from Veh mice (n=4). q<0.1 considered significant.

The effect of E2 on driving differential clustering continued after switching to HFD, as shown by aggregate data on D23 and D27. E-E groups clustered separately from V-V (PERMANOVA, q=0.003, **Figure 6A**), although *α*-diversity was not affected by E2. These changes were mostly due to increases in relative abundances of *Escherichia. Shigella* and *Akkermansia*, and decreases in that of *Anaerostipes*, *Turicibacter, Lactococcus, Lactobacillus, Blautia* and *Clostridium_ IV/XIVa/XIVb* (**Table 3** and **Figure 6B**).

Similarly, E2 altered both *α*-diversity and *β*-diversity on D32 and D39 (aggregate data) in HFD-fed mice. E-E groups had decreased *α*-diversity compared to V-V mice (ANOVA, p=0.035, **Figure 5A**). On the community level, E-E mice clustered differently from V-E_A_ (PERMANOVA, q=0.042), but not V-V (**Figure 6A**), suggesting that the effect of E2 on gut microbiota started to attenuate around 4^th^ week of E2 implant. During this time, E-E mice had increased relative *Escherichia.Shigella* and *Parasutterella* abundances compared to V-V groups. In contrast, E-E mice had lower relative abundances of *Acetanaerobacterium*, *Anaerotruncus, Clostridium_XVIII/XIV, Turicibacter, Enterococcus, Lactobacillus*, and *Romboutsia*, compared to V-V mice, on D32 and D39 (**Table 3** and **Figure 6B**). Interestingly, the E2-induced increase in the relative abundance of *Akkermansia* observed on the D23 and D27 of E2 treatment did not persist at D32 or D39, suggesting that HFD increased *Akkermansia* in both groups, eliminating the difference between the E2 and Veh groups.

## Discussion

In the present study, we tested the hypothesis that the gut microbiota mediates some of the protective effects of estrogens on energy metabolism in female mice. Using adult female mice, we investigated the metabolic outcome of cohousing and transfer of the gut microbiota from estrogen-treated lean donors to estradiol-deficient HFD-fed mice. The present findings extend previous reports that estradiol treatment protects ovariectomized HFD-fed mice from hyperphagia, obesity, and hyperglycemia and improves active and basal energy expenditure (5–9, 44). Contrary to our hypothesis, we found that FMT from E2-treated lean donors was not sufficient to transfer a lean phenotype and metabolic benefits to ovariectomized recipients fed a HFD, although a tendency towards improved blood glucose levels was present.

In an effort to maximize the potential effects of gut microbiota on metabolism in female mice, we supplemented the FMT from lean E2-treated mice with *A. muciniphila*, a bacterial species previously reported to alleviate metabolic insults in male rodents, men, and women (17–19, 29, 31–33, 63), but *c.f (* 64 *).* In addition, we have previously identified that the relative abundance of *Akkermansia* increases in E2-treated female mice compared to ovariectomized controls and is inversely correlated to weight gain and fat mass (44). In the current study, while an FMT supplemented with *A. muciniphila* altered the relative abundance of many gut microbial species, surprisingly, it did not improve metabolic health but seemed to negatively affect blood glucose in ovariectomized mice fed HFD. These novel findings in female mice suggest that a transplant of fecal microbiota supplemented with *A. muciniphila*, under the present experimental conditions, is not sufficient to transfer the metabolic phenotype and could aggravate some HFD-induced insults. Although the colonization of many microbes *via* FMT persisted, introduction of HFD profoundly and acutely increased *Akkermansia* in all treatment groups. These data suggest that enriching FMT from lean mice with *A. muciniphila* disrupts glucose homeostasis. Alternatively, while the supplementation with only *A. muciniphila* could have beneficial effects on metabolic health, addition of *A. muciniphila* in feces from E2-treated female mice, that already contain this microbe, may exert detrimental effects by disrupting the microbial community homeostasis.

The different effects of *Akkermansia* between the current study and previous findings may be due to sex differences in the effects of *Akkermansia* in the mammalian gut. Neither HFD nor a high-fat high-sugar diet induced *Akkermansia* in male mice (17, 31, 46). In dramatic contrast, a week of HFD in female mice elicited a robust increase in the relative abundance of *Akkermansia* as reported here and previously (7). *Akkermansia* uses mucin as its sole nutrient source, which is an integral component of the gastrointestinal mucosa layer (25, 28). Sex differences also exist in intestinal mucin in the manifestation of obesity. In male mucin2 knockout mice, HFD-induced obesity and hyperglycemia from alcohol-induced hepatosteatosis was attenuated (65, 66), while female mucin2-deficient mice had exacerbated glucose tolerance and were not protected from obesity. Thus, a sex difference in the nutrient source for *Akkermansia* could lead to differences in their abundance and function between males and females (Hartmann, 2016). In addition, but not mutually exclusive of this possible sex difference, the disparate outcomes between the present findings and previous studies could be due to other differences in experimental design, including use of cecal vs. fecal material transplant, use of live vs. killed *A. muciniphila* cells, the number and frequency of FMT gavages, and the presence or absence of cohousing donors (14, 17, 19). It is important to note that gut microbiota elicits a variety of responses based on the factors contributing to metabolic disorders. For example, a transfer of healthy microbiota attenuated body weight gain and improved insulin response in PCOS models of female mice) (60), whereas did not prevent ovariectomy-dependent obesity (67). Most importantly, the present findings provide a compelling justification for further investigation of sex differences in basic and clinical studies in the function of gut microbiota in metabolic health.

The differences between the present and previous studies could also be due to the use of antibiotics for initial depletion of the native gut microbiota prior to *A. muciniphila* gavage in the current study unlike in previous studies (17, 19). Antibiotics interact with estrogens (34, 68, 69), primarily by altering the composition of the gut microbiota and E2 metabolism. In support, the mammalian gut is ubiquitously colonized by microbes that produce the steroid-metabolizing enzyme, *β*-glucuronidase, which is responsible for the deconjugation and reuptake of E2 in enterohepatic circulation (34, 70–72). *β*-glucuronidase activity has been observed in *Bacteroides* and *Ruminococcus* (73), which were decreased by antibiotics in the present study. Antibiotic treatment decreases *β*-glucuronidase and increases excretion of conjugated estrogens in feces (71, 74). Thus, the reuptake and availability of E2 was likely diminished by antibiotic administration in the first two weeks of the study, possibly *via* depletion of this E2-metabolizing microbial community. Although the goal of using the lean-FMT background for *A. muciniphila* in the current study was to replenish the beneficial microbial community depleted by antibiotics, it is possible that the HFD intake after the antibiotic treatment permanently disrupted the healthy microbial ecosystem, potentially resulting in an increased mucus production that may have resulted in an overwhelming increase of *Akkermansia*. In this context, it is of interest to note that blooms of *Akkermansia* spp., in human have been described in antibiotic-treated male patients without apparent negative health effects (75).

In the current study, FMT from E2-treated lean mice supplemented with *A. muciniphila* caused hyperglycemia and an increased trend towards body weight gain, suggesting A. *muciniphila* supplementation has a detrimental effect on metabolic health in female mice under the used conditions. The increase in *Akkermansia* following gavage with A. *muciniphila*-supplemented FMT was accompanied by a parallel decrease in *Parasutterella* and *Bacteroides. Parasutterella* is decreased in prediabetic rats and pregnant women with gestational diabetes mellitus and this decrease is associated with a decrease in short chain fatty acid levels (76–78). Similarly, administration of *Bacteroides* attenuates HFD-induced obesity, hyperglycemia and insulin resistance in rats (79). Taken together with the present study, these findings suggest that a decrease in these microbes contributes to *Akkermansia*-dependent impairment in metabolism in females.

In summary, FMT from lean E2-treated mice mildly improves blood glucose levels in female mice fed a HFD, but does not protect from obesity. However, enriching FMT from lean mice with *A. muciniphila* disrupts glucose homeostasis in the present model. Based on the existing evidence of beneficial effects of *A. muciniphila* on metabolic health, mostly observed in male animal models, clinical trials using an *A. muciniphila* supplement in humans have been completed (18, 19), where safety and efficacy of *A. muciniphila* supplementation have been shown in both female and male subjects. It will be important to determine if the present findings in estradiol-deficient HFD female mice extrapolate to humans. Moreover, it is critical that future studies investigate sex differences in host-*Akkermansia* interactions regarding metabolic health.

## Data availability statement

The datasets presented in this study can be found in online repositories. The names of the repository/repositories and accession number(s) can be found below: <b><br>https://www.ncbi.nlm.nih.gov/, SAMN30108647 C01 C01 mouse metagenome 1441287 SAMN30108648 C12 C12 mouse metagenome 1441287 SAMN30108649 C13 C13 mouse metagenome 1441287 SAMN30108650 C02 C02 mouse metagenome 1441287 SAMN30108651 C03 C03 mouse metagenome 1441287 SAMN30108652 C04 C04 mouse metagenome 1441287 SAMN30108653 C07 C07 mouse metagenome 1441287 SAMN30108654 C08 C08 mouse metagenome 1441287 SAMN30108655 EE19 EE19 mouse metagenome 1441287 SAMN30108656 EE21 EE21 mouse metagenome 1441287 SAMN30108657 EE23 EE23 mouse metagenome 1441287 SAMN30108658 EE25 EE25 mouse metagenome 1441287 SAMN30108659 EE27 EE27 mouse metagenome 1441287 SAMN30108660 D01EEM10 D01EEM10 mouse metagenome 1441287 SAMN30108661 D14EEM10 D14EEM10 mouse metagenome 1441287 SAMN30108662 D17EEM10 D17EEM10 mouse metagenome 1441287 SAMN30108663 D23EEM10 D23EEM10 mouse metagenome 1441287 SAMN30108664 D27EEM10 D27EEM10 mouse metagenome 1441287 SAMN30108665 D32EEM10 D32EEM10 mouse metagenome 1441287 SAMN30108666 D39EEM10 D39EEM10 mouse metagenome 1441287 SAMN30108667 D07EEM10 D07EEM10 mouse metagenome 1441287 SAMN30108668 D01EEM11 D01EEM11 mouse metagenome 1441287 SAMN30108669 D14EEM11 D14EEM11 mouse metagenome 1441287 SAMN30108670 D17EEM11 D17EEM11 mouse metagenome 1441287 SAMN30108671 D23EEM11 D23EEM11 mouse metagenome 1441287 SAMN30108672 D27EEM11 D27EEM11 mouse metagenome 1441287 SAMN30108673 D32EEM11 D32EEM11 mouse metagenome 1441287 SAMN30108674 D39EEM11 D39EEM11 mouse metagenome 1441287 SAMN30108675 D07EEM11 D07EEM11 mouse metagenome 1441287 SAMN30108676 D01EEM12 D01EEM12 mouse metagenome 1441287 SAMN30108677 D14EEM12 D14EEM12 mouse metagenome 1441287 SAMN30108678 D17EEM12 D17EEM12 mouse metagenome 1441287 SAMN30108679 D23EEM12 D23EEM12 mouse metagenome 1441287 SAMN30108680 D27EEM12 D27EEM12 mouse metagenome 1441287 SAMN30108681 D32EEM12 D32EEM12 mouse metagenome 1441287 SAMN30108682 D39EEM12 D39EEM12 mouse metagenome 1441287 SAMN30108683 D07EEM12 D07EEM12 mouse metagenome 1441287 SAMN30108684 D01VVM01 D01VVM01 mouse metagenome 1441287 SAMN30108685 D14VVM01 D14VVM01 mouse metagenome 1441287 SAMN30108686 D17VVM01 D17VVM01 mouse metagenome 1441287 SAMN30108687 D23VVM01 D23VVM01 mouse metagenome 1441287 SAMN30108688 D27VVM01 D27VVM01 mouse metagenome 1441287 SAMN30108689 D32VVM01 D32VVM01 mouse metagenome 1441287 SAMN30108690 D39VVM01 D39VVM01 mouse metagenome 1441287 SAMN30108691 D07VVM01 D07VVM01 mouse metagenome 1441287 SAMN30108692 D14VVM02 D14VVM02 mouse metagenome 1441287 SAMN30108693 D17VVM02 D17VVM02 mouse metagenome 1441287 SAMN30108694 D23VVM02 D23VVM02 mouse metagenome 1441287 SAMN30108695 D27VVM02 D27VVM02 mouse metagenome 1441287 SAMN30108696 D32VVM02 D32VVM02 mouse metagenome 1441287 SAMN30108697 D39VVM02 D39VVM02 mouse metagenome 1441287 SAMN30108698 D07VVM02 D07VVM02 mouse metagenome 1441287 SAMN30108699 D01VVM03 D01VVM03 mouse metagenome 1441287 SAMN30108700 D14VVM03 D14VVM03 mouse metagenome 1441287 SAMN30108701 D17VVM03 D17VVM03 mouse metagenome 1441287 SAMN30108702 D23VVM03 D23VVM03 mouse metagenome 1441287 SAMN30108703 D27VVM03 D27VVM03 mouse metagenome 1441287 SAMN30108704 D32VVM03 D32VVM03 mouse metagenome 1441287 SAMN30108705 D39VVM03 D39VVM03 mouse metagenome 1441287 SAMN30108706 D07VVM03 D07VVM03 mouse metagenome 1441287 SAMN30108707 D01VVM04 D01VVM04 mouse metagenome 1441287 SAMN30108708 D14VVM04 D14VVM04 mouse metagenome 1441287 SAMN30108709 D17VVM04 D17VVM04 mouse metagenome 1441287 SAMN30108710 D23VVM04 D23VVM04 mouse metagenome 1441287 SAMN30108711 D27VVM04 D27VVM04 mouse metagenome 1441287 SAMN30108712 D32VVM04 D32VVM04 mouse metagenome 1441287 SAMN30108713 D39VVM04 D39VVM04 mouse metagenome 1441287 SAMN30108714 D07VVM04 D07VVM04 mouse metagenome 1441287 SAMN30108715 D01VAM05 D01VAM05 mouse metagenome 1441287 SAMN30108716 D14VAM05 D14VAM05 mouse metagenome 1441287 SAMN30108717 D17VAM05 D17VAM05 mouse metagenome 1441287 SAMN30108718 D23VAM05 D23VAM05 mouse metagenome 1441287 SAMN30108719 D27VAM05 D27VAM05 mouse metagenome 1441287 SAMN30108720 D32VAM05 D32VAM05 mouse metagenome 1441287 SAMN30108721 D39VAM05 D39VAM05 mouse metagenome 1441287 SAMN30108722 D07VAM05 D07VAM05 mouse metagenome 1441287 SAMN30108723 D01VAM06 D01VAM06 mouse metagenome 1441287 SAMN30108724 D14VAM06 D14VAM06 mouse metagenome 1441287 SAMN30108725 D17VAM06 D17VAM06 mouse metagenome 1441287 SAMN30108726 D27VAM06 D27VAM06 mouse metagenome 1441287 SAMN30108727 D32VAM06 D32VAM06 mouse metagenome 1441287 SAMN30108728 D39VAM06 D39VAM06 mouse metagenome 1441287 SAMN30108729 D07VAM06 D07VAM06 mouse metagenome 1441287 SAMN30108730 D01VAM07 D01VAM07 mouse metagenome 1441287 SAMN30108731 D14VAM07 D14VAM07 mouse metagenome 1441287 SAMN30108732 D17VAM07 D17VAM07 mouse metagenome 1441287 SAMN30108733 D23VAM07 D23VAM07 mouse metagenome 1441287 SAMN30108734 D27VAM07 D27VAM07 mouse metagenome 1441287 SAMN30108735 D32VAM07 D32VAM07 mouse metagenome 1441287 SAMN30108736 D39VAM07 D39VAM07 mouse metagenome 1441287 SAMN30108737 D07VAM07 D07VAM07 mouse metagenome 1441287 SAMN30108738 D01VAM08 D01VAM08 mouse metagenome 1441287 SAMN30108739 D14VAM08 D14VAM08 mouse metagenome 1441287 SAMN30108740 D17VAM08 D17VAM08 mouse metagenome 1441287 SAMN30108741 D23VAM08 D23VAM08 mouse metagenome 1441287 SAMN30108742 D27VAM08 D27VAM08 mouse metagenome 1441287 SAMN30108743 D32VAM08 D32VAM08 mouse metagenome 1441287 SAMN30108744 D39VAM08 D39VAM08 mouse metagenome 1441287 SAMN30108745 D07VAM08 D07VAM08 mouse metagenome 1441287 SAMN30108746 D01EEM09 D01EEM09 mouse metagenome 1441287 SAMN30108747 D14EEM09 D14EEM09 mouse metagenome 1441287 SAMN30108748 D17EEM09 D17EEM09 mouse metagenome 1441287 SAMN30108749 D23EEM09 D23EEM09 mouse metagenome 1441287 SAMN30108750 D27EEM09 D27EEM09 mouse metagenome 1441287 SAMN30108751 D32EEM09 D32EEM09 mouse metagenome 1441287 SAMN30108752 D39EEM09 D39EEM09 mouse metagenome 1441287 SAMN30108753 D07EEM09 D07EEM09 mouse metagenome 1441287 SAMN30108754 VA19 VA19 mouse metagenome 1441287 SAMN30108755 VA21 VA21 mouse metagenome 1441287 SAMN30108756 VA23 VA23 mouse metagenome 1441287 SAMN30108757 VA25 VA25 mouse metagenome 1441287 SAMN30108758 VA27 VA27 mouse metagenome 1441287 SAMN30108759 VV19 VV19 mouse metagenome 1441287 SAMN30108760 VV21 VV21 mouse metagenome 1441287 SAMN30108761 VV23 VV23 mouse metagenome 1441287 SAMN30108762 VV25 VV25 mouse metagenome 1441287 SAMN30108763 VV27 VV27 mouse metagenome 1441287.

## Ethics statement

All procedures were approved by the Institutional Animal Care and Use Committees of University of Massachusetts Chan School and Wellesley College and performed in accordance with National Institutes of Health Animal Care and Use Guidelines.

## Author contributions

Each author has made substantial contributions to the work: Conceptualization; formal analysis; writing—original draft; supervision, review and editing: KA, DW, MG, WV, BM, JK and MT. Methodology, review and editing: KA, RF, DW, MG, LT, DZ, and XH. Formal Analysis: KA, RF, DW, and MG, Project administration and funding acquisition: KA, WV, BM, JK, and MT. All authors have read and agreed to the published version of the manuscript.

## Funding

This work was funded in part by NIH 5U24DK076169-13 Subaward # 30835-64 (KDA), SIAM Gravitation Grant 024.002.002 of the Netherlands Organization for Scientific Research (WMdV), NIH DK125407 and DK109677 (BAM), NIH 5U2C-DK093000 (JKK), and NIH DK61935 and Wellesley College Jenkins Distinguished Chair in Neuroscience Funds (MJT). Part of this study was performed at the National Mouse Metabolic Phenotyping Center (MMPC) at University of Massachusetts Chan Medical School.

## Conflict of interest

WV is co-founder and has stock in The Akkermansia Company, and BM is a coinventor on a patent application PGT/US 18/42116 emanating, in part, from the findings described herein, and along with her respective academic institution, stands to gain financially through potential commercialization outcomes resulting from activities associated with the licensing of that intellectual property.

The remaining authors declare that the research was conducted in the absence of any commercial or financial relationships that could be construed as a potential conflict of interest.

## Publisher’s note

All claims expressed in this article are solely those of the authors and do not necessarily represent those of their affiliated organizations, or those of the publisher, the editors and the reviewers. Any product that may be evaluated in this article, or claim that may be made by its manufacturer, is not guaranteed or endorsed by the publisher.

## Supplementary material

Supplementary Figure 1 Hourly measures of **(A)** food intake and **(B)** physical activity in HFD-fed adult female mice in metabolic cages on days 18–21. E-E, E2-treated mice receiving FMT from E2 mice (n=4); V-V, mice with Veh implants receiving FMT from Veh mice (n=4); V-E_A_, mice with Veh implants receiving FMT from E2-treated mice supplemented with *A. muciniphila* (n=4).

Supplementary Figure 2 Confirmation of *Akkermansia* in fecal microbiota transplant (FMT) gavage samples. **(A)** Microbiota taxa relative abundance at genus level in FMT samples, pooled from all donors of the same treatment group (E2 donors n=8; Veh donors n=4). **(B)** Principal component plot showing weighted Unifrac distance for the FMT samples. E-FMT: FMT from E2-treated donors; V-FMT: FMT from Veh donors.
