# Supplementary figures and images for: Differential effects of Akkermansia-enriched fecal microbiota transplant on energy balance in female mice on high-fat diet
Source: Front Endocrinol (Lausanne). 2022 Oct 27;13:1010806. doi: 10.3389/fendo.2022.1010806 (PMC9647077; doi:10.3389/fendo.2022.1010806)

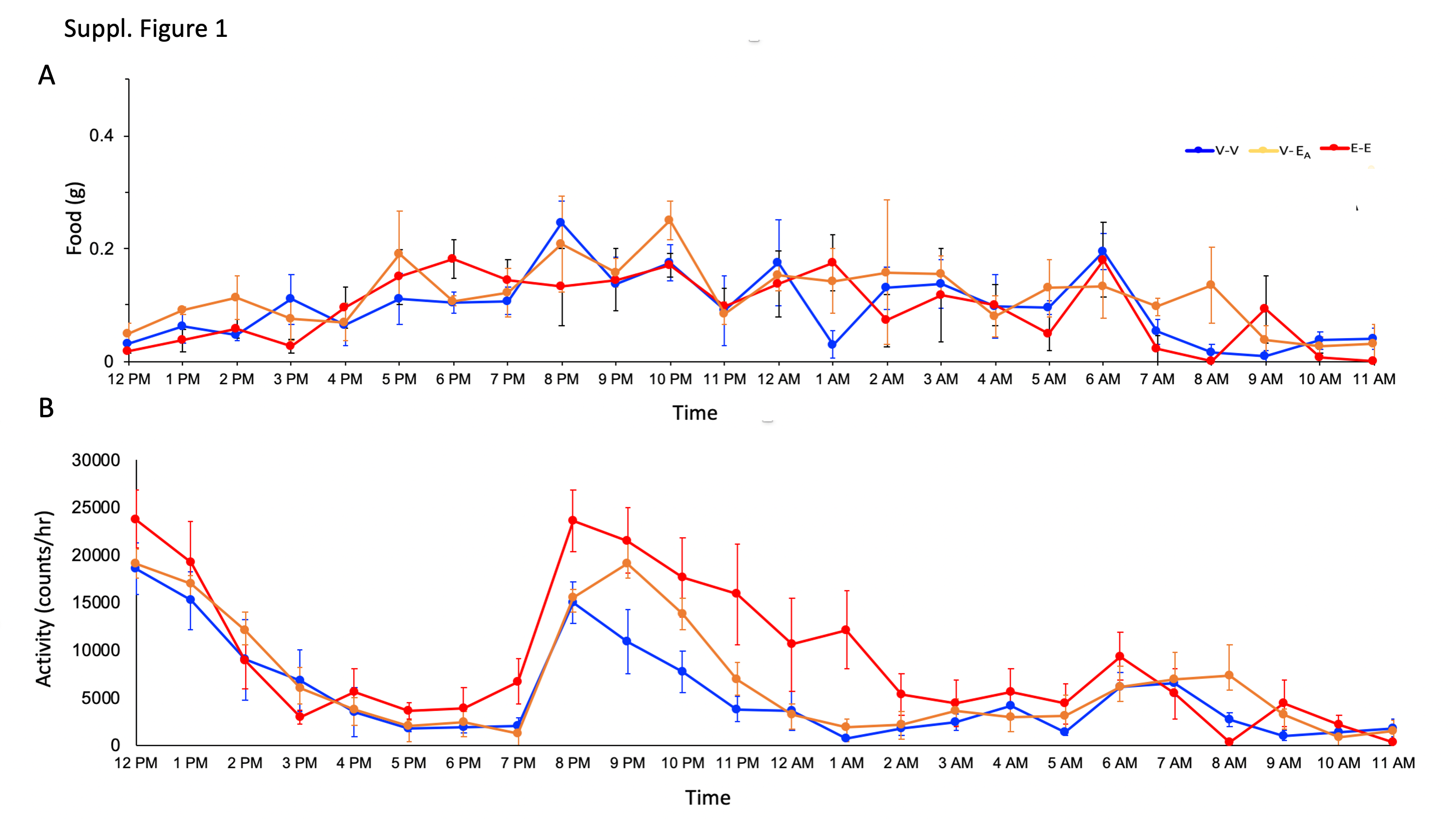

Supplement: Supplementary Figure 1 — Hourly measures of (A) food intake and (B) physical activity in HFD-fed adult female mice in metabolic cages on days 18–21. E-E, E2-treated mice receiving FMT from E2 mice (n=4); V-V, mice with Veh implants receiving FMT from Veh mice (n=4); V-EA, mice with Veh implants receiving FMT from E2-treated mice supplemented with A. muciniphila (n=4). [file Image_1.tiff]

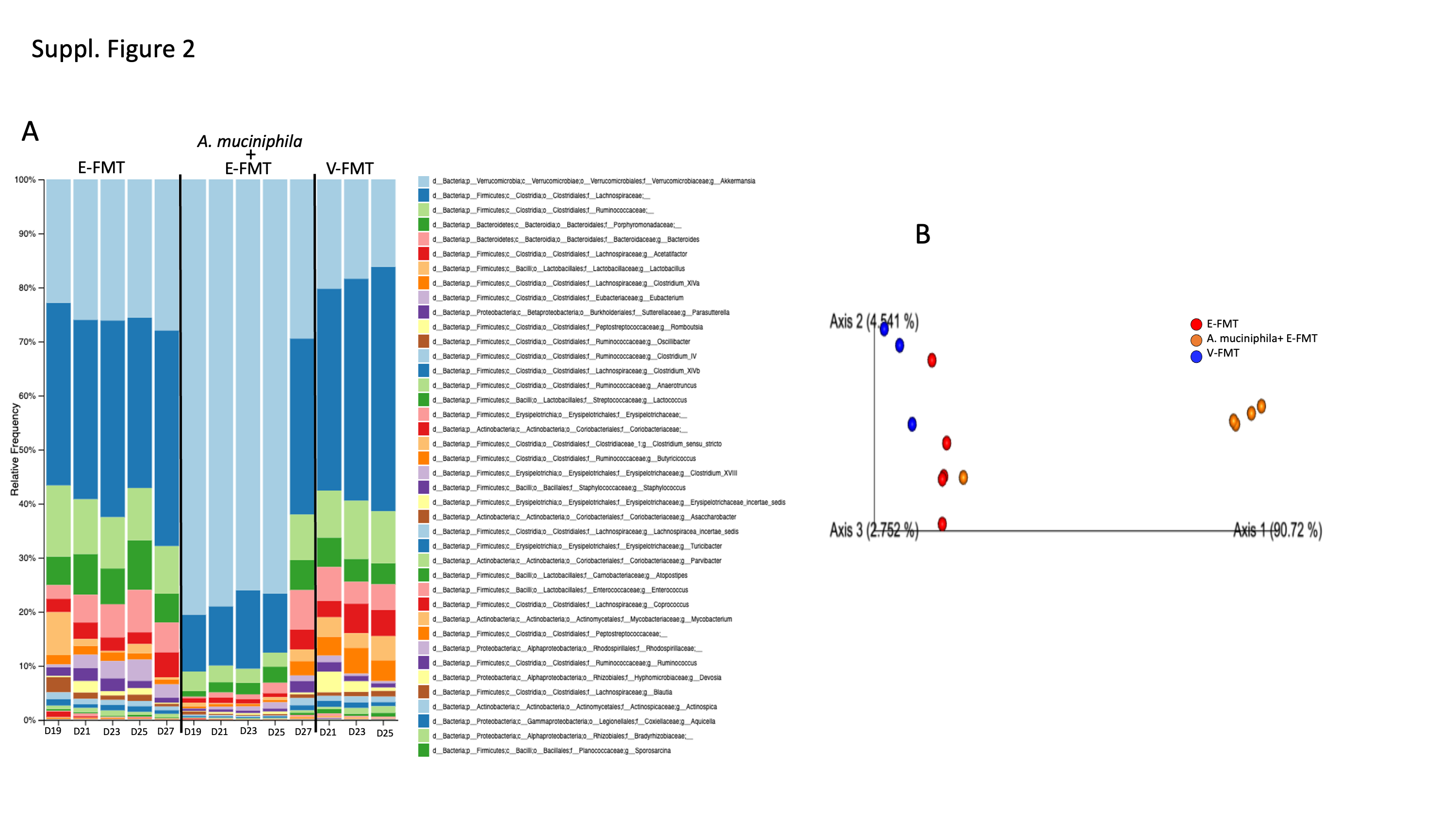

Supplement: Supplementary Figure 2 — Confirmation of Akkermansia in fecal microbiota transplant (FMT) gavage samples. (A) Microbiota taxa relative abundance at genus level in FMT samples, pooled from all donors of the same treatment group (E2 donors n=8; Veh donors n=4). (B) Principal component plot showing weighted Unifrac distance for the FMT samples. E-FMT: FMT from E2-treated donors; V-FMT: FMT from Veh donors. [file Image_2.tiff]
